# Car Sanitation in Texas

**Published:** 1902-04

**Authors:** 


### TEXAS MEDICAL JOURNAL.

AUSTIN, TEXAS.

A MONTHLY JOURNAL OF MEDICINE AND SURGERY.

EDITED AND PUBLISHED BY
F. E. DANIEL, M. D.

ASSOCIATE EDITOR:
WITTEN BOOTH RUSS, M. D.,
San Antonio, Texas.

Published Monthly at Austin, Texas. Subscription price $1.00 a year in advance.

Eastern Representative: John Guy Monihan, St. Paul Building, 220 Broadway, New York City.

Official organ of the State Association of Health Officers, the West Texas Medical Association, the Houston District Medical Association, the Austin District Medical Society, the Brazos Valley Medical Association, the Galveston County Medical Society, and several others.

### CAR SANITATION IN TEXAS.

A movement is being inaugurated to cause the disinfection of sleeping cars and day coaches on all the railroads in Texas. It is yet inchoate, but in a conference with State Health Officer Tabor we learn that it will be instituted at an early date, as soon as certain details shall have been determined. First, he is to have an opinion from the Attorney General as to his authority in the matter and the extent of it, and whether the State shall pay for it, or whether or not the railroad companies shall do so. It seems clear to my mind that under the provisions of the quarantine law, (the only "health" law we have in Texas), the State Health Officer has authority to compel the railroad companies to disinfect their cars and to keep them in good sanitary condition, and that they cannot expect the State, or any body else, to pay for their sanitary policing. But—I am not the Attorney General. When the question of authority is determined it will be necessary to arrange the details of the process, and appoint inspectors to see that the cleansing and disinfection are properly carried out,—the adoption of some method of fumigation, etc. The traveling public are becoming aroused to a sense of the danger lurking in upholstered and curtained coaches, and the Travelers' Protective Association—the drummers— made the first protest. Attracted by the mild climate of Texas, hundreds, yes, thousands of consumptives drift into the State every winter, and many return to their northern homes in spring. Hence, during the winter and spring months, especially, the travel of this class on Texas railroads is very heavy Mr. D. A. Michaux, of Houston, member of the Travelers' Protective Association and chairman of its National Committee on Railroads, came to Austin and had an interview with Dr. Tabor, getting his views on the subject, and then wrote and published in the Texas papers "An Open Letter to Dr. Tabor," specifically setting forth the danger, and the drummers' appreciation of it,—of occupying berths or seats, perhaps just vacated by some consumptive, (to say nothing of other contagious diseases, as smallpox, diphtheria, or scarlet fever, or measles), in cars where, as is too frequently the case, passengers expectorate on the floor, or cough and spatter the sputa on the curtains in the sleepers, or on the blankets or sheets, and in the second-class coaches, on the hot stoves, etc. The drummers' association, through him, appeal to Dr. Tabor as the State's sole sanitary authority, for measures of relief from this very great danger,—a nuisance and a constant menace to the lives of the traveling public. The public generally, however, do not appreciate the danger. The majority are totally ignorant of it. Because consumption is not as acutely contagious as are other diseases mentioned, and is not as "catching," they do not realize its terrors. It is as much as one's life is worth to travel in a Texas sleeper in cold weather, for, in addition to the danger of contagion, the cars are not ventilated, but shut up almost air tight and heated to suffocation. In a crowded car, thus closed and heated, every passenger breathes the poisonous atmosphere which has passed through the lungs of every other passenger. This is a nuisance that ought to be at once abated. The State owes it to the public to protect them. It is a sanitary reform pressingly demanded. I hope and believe that Dr. Tabor, who has proven himself to be an efficient and progressive sanitarian, will push it to a speedy consummation. It should not stop with cleansing and disinfection, but the railroads should be required to provide sleepers and other coaches equipped with seats of rattan, cane, or nickle wire, and berths of woven wire, and efficient means for ventilation; and the filth-catching, microbe-breeding, death-dealing old curtains hung before each berth for protection and privacy, but which afford neither, should be replaced by screens or curtains of canvas or something better, at any rate. It seems to me that this latter requirement could and should be enforced by the Railroad Commission, in the event that the State Health Officer is not authorized to go so far.

